# Evaluation of stiffness values of normal uterine myometrium by 3T magnetic resonance elastography

**DOI:** 10.3389/fmed.2026.1743585

**Published:** 2026-02-04

**Authors:** Levent Karakaş, Süheyl Poçan, Şükrü Şahin

**Affiliations:** 1 Department of Radiology, Gaziosmanpaşa Training and Research Hospital, İstanbul, Türkiye; 2 Department of Radiology, İstanbul Nişantaşı University and BHT CLINIC İstanbul Tema Hospital, İstanbul, Türkiye; 3 Department of Radiology, Elazığ Fethi Sekin City Hospital, Elazığ, Türkiye

**Keywords:** 3 tesla magnetic resonance imaging, age, magnetic resonance elastography, menopause, myometrium, stiffness

## Abstract

**Background:**

There is not enough radiologic data in the literature for the quantitative value of uterine stiffness and this study aims to contribute to the literature in this field.

**Objective:**

To evaluate the feasibility of measuring uterine myometrial stiffness (UMS) using 3T magnetic resonance elastography (MRE) in women with radiologically normal uteri and to explore potential associations with demographic and anatomical variables.

**Methods:**

Data on demographics, including age, BMI, obstetric history (gravida and parity), menopausal status, and oral contraceptive use, were gathered. Uterine measurements, such as the long axis, transverse diameter, anteroposterior diameter, and volume, were obtained using 3T MR imaging. UMS (in Pascals) was quantified using MRE, and its associations with various factors were assessed. Values are reported as mean ± standard deviation (SD).

**Results:**

The study enrolled 48 women with a mean age of 37.54 ± 10.47 years. The average UMS was 2714 ± 300 Pa. A strong positive correlation was found between age and UMS (*r* = 0.814, *p* < 0.001), whereas no significant associations were found with BMI, uterine dimensions, gravida, or parity. UMS was higher in postmenopausal women than in women of reproductive age (*p* < 0.001). However, no statistically significant differences were observed between oral contraceptive users and non-users in this limited sample.

**Conclusion:**

This feasibility study demonstrates the practicality of assessing uterine myometrial stiffness using 3T MRE and provides exploratory estimates in radiologically normal uteri. Larger prospective studies are required before normative ranges can be established.

## 1 Introduction

The uterus, a central organ of the female reproductive system, plays a crucial role in menstruation, implantation, and pregnancy (1). Its unique structural and functional characteristics are essential for reproductive health, and alterations in uterine tissue properties are often associated with pathological conditions such as adenomyosis, fibroids, endometriosis, and malignancies (1, 2). Traditional methods for evaluating uterine abnormalities, such as biopsies or surgeries, are invasive and carry risks. This has led to growing interest in noninvasive imaging techniques, with elastography showing promise for assessing tissue stiffness. Understanding uterine stiffness and its variations may provide valuable insights into these conditions, aiding in diagnosis and treatment planning.

Measuring the stiffness of gynecological organs using elastography is utilized in various medical fields, including obstetrics and gynecology, particularly for diagnosing and monitoring benign and malignant uterine pathologies, pregnancy monitoring, postpartum evaluation of the uterus, and assisted reproductive techniques (3–15). Ultrasonography-based elastographic methods, such as shear wave elastography (SWE), strain ratio elastography, and acoustic radiation force impulse, are frequently used for this purpose (9, 16, 17). However, these approaches have some limitations, including operator dependence, restricted imaging depth, and variability of results.

As an alternative, magnetic resonance elastography (MRE), a phase-contrast magnetic resonance imaging (MRI) technique, offers distinct advantages in evaluating soft tissue elasticity, providing high reproducibility, operator independence, and comprehensive organ assessment (18). Owing to its radiation-free nature and the aforementioned advantages, MRE has recently gained more attention in obstetric and gynecologic practices and research. Unfortunately, both ultrasonographic elastographic techniques and MRE share a significant limitation: the lack of published reference values for gynecological organs (9). Establishing reference stiffness values for the uterus in patients with a normal uterus on pelvic MRI is essential for differentiating physiological from pathological conditions; however, such data are limited in the literature. Additionally, the influence of demographic and anatomical factors, such as age, menstrual phase, uterine dimensions, and body mass index (BMI), on uterine stiffness remains unclear.

To address this gap in the literature, we aimed to define the normal range of uterine myometrial stiffness (UMS) in patients with a uterus that appears normal in signal and morphology on pelvic MRI using 3 Tesla (T) MRE and to investigate potential relationships between UMS and demographic, uterine anatomical, and clinical parameters. This exploratory feasibility study aims to provide preliminary stiffness estimates in radiologically normal uteri and to inform the design of future larger studies evaluating the clinical utility of uterine MRE.

## 2 Materials and methods

### 2.1 Study population and design

This retrospective study was conducted in the Department of Radiology at BHT CLINIC Istanbul Tema Hospital. The study was performed in October 2025 by retrospectively reviewing patients who had undergone lower abdominal (pelvic) MRI between January 2021 and September 2024 for indications unrelated to gynecologic pathology or conditions indirectly affecting the uterus and other gynecologic organs, and whose uteruses were found to be normal. Patients with normal uterine morphology and signal characteristics on routine MRI sequence images constituted the study population. Women with radiologically normal uteri were referred to as healthy women or individuals in this study.

This retrospective study included 48 women with the following characteristics: age > 18 years, undergoing pelvic MRI for reasons other than gynecological abnormalities, having a normal uterus, and not being pregnant. Women with a history of uterine surgery, trauma, gynecological pathology, or MRI findings such as fibroids, adenomyosis, or other uterine abnormalities, as well as those who were menstruating at the time of MRI or who had given birth or experienced a miscarriage within the past year, were excluded. Additionally, patients with chronic diseases requiring regular medication or any condition affecting the uterus were excluded.

In the center where the study was performed, elastography examination is routinely performed in abdominal MRI procedures; this procedure, which is performed as an additional sequence in addition to the classical images, does not cause a significant increase in the acquisition time or additional cost to the hospital. In addition to the sequence images obtained in routine conventional pelvic MRI examination, the raw data of the elastography examination performed in the center where our study was performed were not routinely given to the patients as images, but were stored in a way that could be used radiologically. In addition to the sequence images obtained in routine conventional pelvic MRI examination, the raw data of the elastography examination performed in the center where our study was performed are not routinely given to the patients as images, but are stored as raw data that can be used radiologically to be used when necessary.

### 2.2 Data gathering and imaging protocols

In December 2024, MRE evaluations and measurements were scanned and collected at the radiological workstation on the images of patients belonging to our study population selected from patients who underwent MRI examination between January 2021 and September 2024 (Supplementary material). Oher data of the patients were obtained from the information management system of our hospital. Demographic and clinical information, including age, BMI, gravida, parity, menopausal status, and oral contraceptive use were recorded. Uterine dimensions (longitudinal, transverse, and anteroposterior measurements) and myometrium stiffness values were obtained using MRI and MRE, respectively. Uterine dimensions were measured on axial MRI images; therefore, the transverse and anteroposterior diameters reflect axial-plane geometry and uterine orientation at the time of imaging, which may result in values that differ from measurements obtained in other imaging planes. Uterine volume was calculated using the formula 0.5236 × longitudinal × transverse × anteroposterior diameter (19).

Imaging was performed using a 3T MRI system equipped with a torso coil (GE 3.0T Signa Architect, General Electric Medical Systems; Milwaukee, WI, United States). The participants underwent imaging after a 6-h fasting period. Routine pelvic MRI protocols included axial and coronal T2-weighted sequences with a slice thickness of 5 mm. For MRE, a pneumatic driver with a membrane of 19 cm was placed suprapubically to generate mechanical waves. MRE data were acquired at a 60 Hz excitation frequency, utilizing axial T2-weighted images for stiffness evaluation (slice thickness: 10 mm; TR/TE: 50 ms / 18.4 msX ms; FOV: 24–42 cm; matrix: 256 × 64).

**FIGURE 1:**
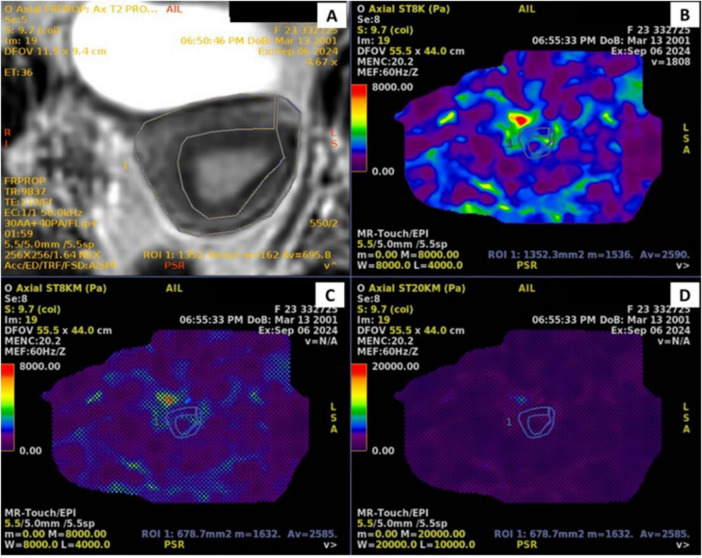
Pelvic MRI of a 30-year-old patient. **(A)** Axial T2 SE. **(B)** Elastography color map. **(C)** Elastography wave map. **(D)** Stiffness map.

Uterine myometrium stiffness was assessed by delineating a region of interest (ROI) on the largest uterine cross-section using fused elastography and T2-weighted images (Figure 1). Each measurement was performed thrice to ensure accuracy and reproducibility. The ROI was drawn to primarily encompass the myometrium while avoiding visible endometrial cavity fluid when present; however, partial-volume inclusion of adjacent endometrium cannot be fully excluded and may contribute to measurement variability.

### 2.3 Outcomes

The primary outcome was to evaluate the feasibility of measuring uterine myometrial stiffness using 3T MRE in women with radiologically normal uteri. The secondary outcome was to examine the relationships between UMS and factors such as age, BMI, uterine dimensions and volume, gravida, parity, menopausal status, and oral contraceptive use. The third outcome was the evaluation of UMS differences by dividing the participants into two groups based on reproductive and postmenopausal status and two groups based on oral contraceptive use (users and non-users).

### 2.4 Statistical analysis

All statistical analyses were performed using IBM SPSS for Windows (version 25.0; IBM Corp., Armonk, NY, United States), with a significance threshold of *p* < 0.05. The normality of continuous variables was assessed using the Kolmogorov-Smirnov test. Descriptive statistics are presented as mean ± standard deviation for continuous variables with normal distribution and frequency (percentage) for categorical variables. Data showing normal distribution were analyzed using Pearson’s correlation test, while ordinal data were assessed using Spearman’s correlation test. Student’s *t*-test was used for group comparisons.

## 3 Results

**FIGURE 2:**
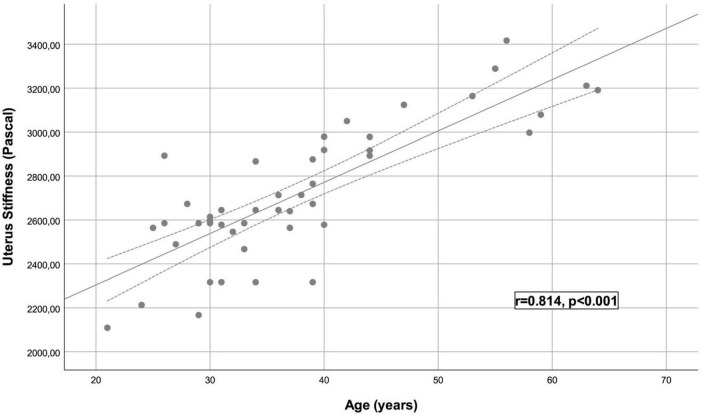
Scatter plot of uterine myometrial stiffness measurement and age.

**FIGURE 3:**
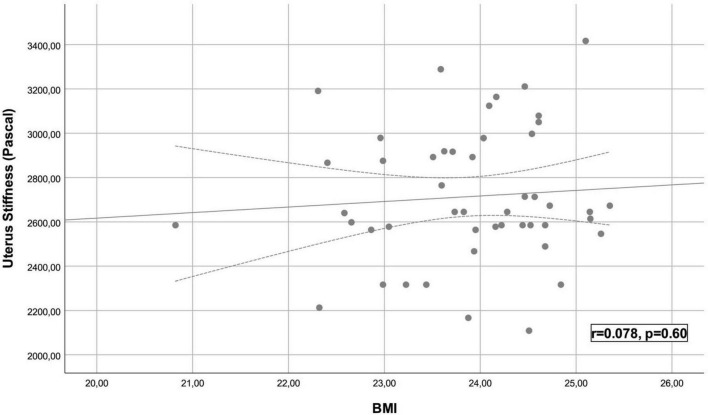
Scatter plot of uterine myometrial stiffnessmeasurement and body mass index.

A total of 48 healthy participants were included in the study, with a mean age of 37.54 ± 10.47 years. The mean BMI was 23.89 ± 0.93 kg/m^2^. The average uterine length was 7.50 ± 0.30 cm, transverse diameter was 2.56 ± 0.21 cm, and anteroposterior diameter was 5.61 ± 0.55 cm. The mean uterine volume was 56.61 ± 9.55 cm^3^. The average UMS was 2714 ± 300 Pa. Statistical analysis revealed a significant and strong positive correlation between age and UMS (*r* = 0.814, *p* < 0.001) (Figure 2). However, no significant correlation was found between UMS and BMI (*r* = 0.078, *p* = 0.600) (Figure 3), longitudinal diameter (*r* = −0.189, *p* = 0.189), transverse diameter (*r* = −0.177, *p* = 0.229), anteroposterior diameter (*r* = −0.219, *p* = 0.136), uterine volume (*r* = −0.262, *p* = 0.072), gravida (*r* = −0.166, *p* = 0.260), or parity (*r* = −0.172, *p* = 0.242) (Table 1).

**TABLE 1:** Correlation between uterine stiffness and various factors.

Variable	Descriptive statistic	*r*	*p*
Age (years)	37.54 ± 10.47	0.814	<0.001
Body mass index (kg/m^2^)	23.89 ± 0.93	0.078	0.600
Longitudinal diameter (cm)	7.50 ± 0.30	−0.189	0.189
Transverse diameter (cm)	2.56 ± 0.21	−0.177	0.229
Anteroposterior diameter (cm)	5.61 ± 0.55	−0.219	0.136
Uterine volume (cmł)	56.61 ± 9.55	−0.262	0.072
Gravida	1.73 ± 1.05	−0.166	0.260
Parity	1.33 ± 1.00	−0.172	0.242
Uterine stiffness (Pascal)	2,714 ± 300.		

Descriptive statistics are given as mean ± standard deviation for normally distributed continuous variables. r: Correlation coefficient for stiffness.

**FIGURE 4:**
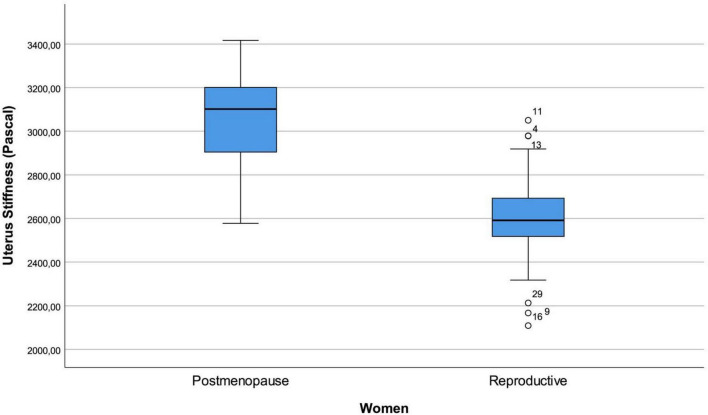
Box plot of uterine myometrial stiffness measurements comparing postmenopausal and reproductive women.

When participants were stratified by menopausal status, uterine myometrial stiffness values differed between women of reproductive age and postmenopausal women (*p* < 0.001) (Figure 4). In contrast, no statistically significant associations were observed between UMS and body mass index, uterine dimensions, gravida, parity, or oral contraceptive use in this limited cohort. These subgroup analyses should be interpreted cautiously, as the study was not powered to detect small-to-moderate differences (Table 2).

**TABLE 2:** The effect of fertility status and oral contraceptive use on uterine stiffness values.

Variable	Uterine stiffness value (Pascal)	*p*
Reproductive	2,598 ± 224	< 0.001
Postmenopausal	3,060 ± 225	
Oral contraceptive users	2,746 ± 274	0.399
Oral contraceptive non-users	2,672 ± 332	

Descriptive statistics are given as mean ± standard deviation for normally distributed continuous variables.

## 4 Discussion

Elastography is an innovative imaging technique that noninvasively evaluates the mechanical properties of organs and tissues. Its potential use in gynecological pathologies and obstetric monitoring has attracted attention in recent years. Elastography has increasingly been applied in obstetric and gynecologic imaging to assess tissue mechanical properties across a range of conditions, including adenomyosis, uterine fibroids, cervical pathology, and reproductive health. Prior studies have demonstrated that tissue stiffness measurements may reflect underlying histological and hormonal changes; however, reported values and influencing factors vary considerably depending on the imaging modality, acquisition parameters, and study population. In this context, magnetic resonance elastography offers potential advantages over ultrasound-based techniques, including operator independence and whole-organ assessment, but data on uterine applications remain limited (1, 3–15, 20). Importantly, discrepancies among prior studies highlight the need for feasibility-oriented investigations using standardized MRE protocols before clinically meaningful reference ranges can be proposed. However, a recent scoping review by Horwood et al. highlighted that one of the significant limitations of the use of these technologies is the lack of published reference values for gynecological organs (9). Establishing normal tissue stiffness values for gynecological organs, such as the uterus, can facilitate the understanding of physiological changes in healthy individuals and the early detection of pathological conditions. Many studies have shown that MRI is a powerful tool for visualizing soft tissues with high resolution, and its ability to capture mechanical properties, such as elasticity and viscosity, is increasingly being explored using techniques such as MRE. Several studies have demonstrated the feasibility of MRE in assessing uterine viscoelasticity and its sensitivity to physiological, regional, and pathological variations (6, 21).

This study aimed to evaluate the feasibility of uterine myometrial stiffness assessment using MRE and to provide exploratory stiffness estimates in healthy women. In the present study, the mean UMS value measured by MRE was 2,714 ± 300 Pa. UMS was significantly and positively correlated only with advanced age. No significant correlations were found between UMS and BMI, uterine longitudinal, transverse, and anteroposterior diameters, uterine volume, gravida, or parity. Postmenopausal women had significantly higher UMS than reproductive-aged women. The use of oral contraceptives did not significantly affect the UMS.

To our knowledge, no previous studies have measured UMS using MRE and investigated related factors in healthy women. Therefore, we discussed the results and findings of studies that investigated uterine stiffness values and the factors affecting these values in healthy women using various methods. In a prospective study, the UMS was evaluated in healthy women using the transabdominal SWE method. The average uterine stiffness was 6.38 kPa, with a median stiffness of 5.61 kPa (range: 2.76–11.31 kPa). When the uterus was fully visible, the stiffness value averaged 7.26 kPa, whereas it decreased to 4.04 kPa when it was only partially visible. Examining factors influencing stiffness, no significant effects of age and BMI were found, but the mode of delivery was a significant determinant. Uterine stiffness was 3.83 kPa in individuals who had cesarean deliveries, 9.17 kPa in those with vaginal deliveries, and 6.35 kPa in individuals who had not given birth. Additionally, the use of oral contraceptives and other clinical parameters did not significantly affect the uterine stiffness (22). In another prospective study conducted by Manchanda et al., the uterine stiffness of 56 healthy women was measured using 2D SWE. The average stiffness of the endometrium in healthy individuals was 25.54 ± 8.56 kPa, and that of the myometrium was 40.24 ± 8.59 kPa. In the cervix, the stiffness value was 18.90 ± 4.22 kPa. This study assessed the effects of the menstrual phase and age on uterine stiffness and found no significant differences among the menstrual phases and age groups. This study did not investigate the effects of other factors, such as BMI, uterine dimensions, parity, pregnancy status, oral contraceptive use, and menopausal status, on uterine stiffness (23). In a cross-sectional study, patients were divided into three groups: normal myometrium, adenomyosis, and fibroid groups. Uterine stiffness was measured using SWE and transvaginal ultrasonography. The shear wave velocity (SWV) values in the adenomyosis and fibroid groups were significantly higher than those in the normal myometrium group; however, there was no significant difference between the adenomyosis and fibroid groups. This study found no significant differences in the SWV values between premenopausal and postmenopausal patients. Additionally, no relationship has been found between uterine volume and SWV (7). Castro et al. presented a detailed observational analysis of cervical stiffness and wave speed as assessed by sonoelastography in 50 healthy women. Multiparous women exhibited higher stiffness and faster wave transmission than nulliparous and primiparous women. Age was another factor influencing stiffness, with the highest values observed in women aged 50–65 years old. However, no significant differences were found in relation to BMI, smoking, or the menstrual cycle phase (24). Seol et al. conducted a prospective, multicenter study aimed at validating cervical elastography as a predictor of spontaneous preterm birth in high- and low-risk pregnancies. Cervical stiffness was measured using a transvaginal ultrasound. Weak but significant correlations were observed between gestational age and elastographic parameters. The stiffness ratio was negatively correlated with gestational age. However, factors such as maternal age, BMI, parity, and uterine artery Doppler indices did not significantly affect elastographic measurements (25). A systematic review evaluated elastography studies focusing on the cervix. Normal cervical stiffness values measured by SWE in healthy individuals vary in the literature, generally reported between 3 and 6 kPa. In patients, malignant lesions typically show higher stiffness values (e.g., 10–15 kPa). The factors affecting stiffness were also examined. Analyses related to age indicated that while some studies reported an increase in cervical stiffness with age, others found no significant changes in cervical stiffness. Postmenopausal periods generally report stiffer cervical tissue (26).

This study contributes to the growing body of literature on tissue stiffness by providing normative data on UMS measured using MRE in healthy individuals. The observed positive correlation between age and UMS aligns with the findings of similar studies on organ elasticity. For instance, studies investigating SS have reported age-related increases in stiffness, likely due to histological and physiological changes in tissue composition over time (27, 28). In the uterus, such changes may include increased fibrotic content, decreased vascularity, and alterations in the extracellular matrix, which are well-documented phenomena in the aging process. This hypothesis is supported by prior research demonstrating age-related changes in uterine tissue architecture, particularly in postmenopausal women, where reduced hormone levels are associated with increased fibrosis and diminished elasticity (24). However, the fact that most of the studies discussed above in healthy women did not find a significant relationship between age and uterine stiffness indicates that more research is needed. The significant difference in UMS between the reproductive and postmenopausal groups underscores the impact of hormonal changes on uterine elasticity. The higher UMS values observed in postmenopausal women likely reflect hormonal deprivation, leading to increased fibrosis and reduced tissue compliance. The lack of a significant correlation between UMS and BMI or uterine volume is consistent with the findings of other elastography studies. Previous research has suggested that while BMI can influence the success of measurements in ultrasound-based techniques owing to wave propagation challenges, it does not significantly impact the stiffness values themselves (23–25). Similarly, uterine volume and dimensions appear to have no measurable effect on stiffness in healthy individuals, suggesting that intrinsic tissue properties are the primary determinants of UMS values. The absence of significantdifferences between oral contraceptive users and non-users suggests that exogenous hormone use does not substantially affect uterine stiffness in the studied population. However, further investigation is warranted to confirm this finding and explore the potential long-term effects of hormonal interventions on uterine elasticity. The finding that gravida and parity did not significantly affect uterine stiffness supports the results of previous studies (24). This situation may be related to the hormonal and mechanical adaptations occurring during pregnancy, which are largely reversible in the postpartum period, and the myometrium and connective tissue structures maintaining homeostatic balance. Additionally, the processes of collagen synthesis and degradation not showing significant differences associated with gravida and parity may explain why uterine stiffness remains unaffected by these factors.

Because menopausal status is intrinsically linked to age, the observed difference in uterine myometrial stiffness between reproductive-age and postmenopausal women should be interpreted as associative rather than causal. The present feasibility study was not designed to disentangle the independent effects of age, hormonal status, and other potential confounders. Accordingly, these findings should be viewed as hypothesis-generating and warrant confirmation in larger, adequately powered prospective studies.

One of the strengths of this study is its focus on healthy individuals, which provides a robust reference for future research. However, this study has several limitations. The relatively small sample size and single-center design may limit the generalizability of the findings. Additionally, the lack of histopathological correlation prevents definitive conclusions regarding the underlying mechanisms driving the increase in UMS with age and menopause. Although the study design excluded participants with known uterine or systemic diseases, undiagnosed conditions could not be entirely ruled out. The absence of a patient group limits the ability to generalize these findings to pathological conditions in humans. Previous studies on the uterus have demonstrated that disease processes, such as fibrosis and chronic inflammation, significantly alter stiffness values (3–15). Future research comparing healthy and diseased uteri using standardized elastography protocols is essential to validate the clinical utility of UMS measurement. The absence of anthropometric data beyond BMI limited our ability to assess the influence of other body composition factors on UMS. Smoking status and physical activity levels were not investigated, which could be considered other relative limitations. Differences between cesarean and normal deliveries were not investigated, although previous studies have reported that they may affect uterine stiffness (22). Finally, no other studies have evaluated UMS in healthy women using MRE. Therefore, the results could not be sufficiently compared with those of other similar studies.

## 5 Conclusion

This feasibility study demonstrates the practicality of assessing uterine myometrial stiffness using 3T magnetic resonance elastography in women with radiologically normal uteri. The findings suggest that age and menopausal status are associated with uterine stiffness measurements; however, these associations should be interpreted cautiously given the exploratory design and limited sample size. Rather than establishing definitive normative values, the present results provide preliminary data that may inform the design of future larger, prospective studies aimed at defining standardized reference ranges and clarifying the clinical utility of uterine MRE in gynecologic imaging.

## Data Availability

The original contributions presented in this study are included in this article/Supplementary material, further inquiries can be directed to the corresponding author.
